# Supplementary material for: Risk factors and prognosis of acute lactation mastitis developing into a breast abscess: A retrospective longitudinal study in China
Source: PLoS One. 2022 Sep 1;17(9):e0273967. doi: 10.1371/journal.pone.0273967 (PMC9436116; doi:10.1371/journal.pone.0273967)
Supplement: Attachment — Submitted filename: Response to Reviewers.docx [file pone.0273967.s004.docx]

**Response to reviewers**

Journal requirements:

When submitting your revision, we need you to address these additional requirements.

1. Please ensure that your manuscript meets PLOS ONE's style requirements, including those for file naming. The PLOS ONE style templates can be found at

https://journals.plos.org/plosone/s/file?id=wjVg/PLOSOne_formatting_sample_main_body.pdf and

<https://journals.plos.org/plosone/s/file?id=ba62/PLOSOne_formatting_sample_title_authors_affiliations.pdf>.

I have changed the style as required by PLOS ONE.

2. We suggest you thoroughly copyedit your manuscript for language usage, spelling, and grammar. If you do not know anyone who can help you do this, you may wish to consider employing a professional scientific editing service.

Whilst you may use any professional scientific editing service of your choice, PLOS has partnered with both American Journal Experts (AJE) and Editage to provide discounted services to PLOS authors. Both organizations have experience helping authors meet PLOS guidelines and can provide language editing, translation, manuscript formatting, and figure formatting to ensure your manuscript meets our submission guidelines. To take advantage of our partnership with AJE, visit the AJE website (http://learn.aje.com/plos/) for a 15% discount off AJE services. To take advantage of our partnership with Editage, visit the Editage website (www.editage.com) and enter referral code PLOSEDIT for a 15% discount off Editage services.  If the PLOS editorial team finds any language issues in text that either AJE or Editage has edited, the service provider will re-edit the text for free.

Upon resubmission, please provide the following:

 The name of the colleague or the details of the professional service that edited your manuscript

A copy of your manuscript showing your changes by either highlighting them or using track changes (uploaded as a *supporting information* file)

A clean copy of the edited manuscript (uploaded as the new *manuscript* file).

My manuscript received language editing services from AME Company. The following figure is the supporting document. If you think my manuscript needs further language edit, please let me know. I will do the language editing through AJE's services. Thanks!

3. Thank you for stating the following in the Funding Section of your manuscript:

“[This research was support](https://mail.163.com/js6/read/readhtml.jsp?mid=219:1tbi2wS-OWB0GFivEgAAsw&userType=browser&font=15&color=064977)ted by the Joint Medical Research Program of Chongqing Municipal Health Commission and Chongqing Science and Technology Bureau（[2020FYYX135](https://mail.163.com/js6/read/readhtml.jsp?mid=219:1tbi2wS-OWB0GFivEgAAsw&userType=browser&font=15&color=064977)）. The funders had no role in study design, data collection and analysis, decision to publish, or preparation of the manuscript.”

We note that you have provided funding information that is not currently declared in your Funding Statement. However, funding information should not appear in the Acknowledgments section or other areas of your manuscript. We will only publish funding information present in the Funding Statement section of the online submission form.

Please remove any funding-related text from the manuscript and let us know how you would like to update your Funding Statement. Currently, your Funding Statement reads as follows:

“Lili Jiang received the award. This research was supportted by the Joint Medical Research Program of Chongqing Municipal Health Commission and Chongqing Science and Technology Bureau（2020FYYX135）. URL：http://wsjkw.cq.gov.cn/.

The funders had no role in study design, data collection and analysis, decision to publish, or preparation of the manuscript.”

Please include your amended statements within your cover letter; we will change the online submission form on your behalf.

I have removed the funding-related statements from the manuscript and amended statements within the cover letter.

4. Please include your full ethics statement in the ‘Methods’ section of your manuscript file. In your statement, please include the full name of the IRB or ethics committee who approved or waived your study, as well as whether or not you obtained informed written or verbal consent. If consent was waived for your study, please include this information in your statement as well.

I have added a full ethics statement in the "method" section of my manuscript.

Additional Editor Comments:

Dear authors,
Thank you for submitting the manuscript to PLOS ONE. According to the reviewers’ comments and my evaluation, the manuscript need careful attention and must be improved according to all the comments.

The Title of the study must indicate the study’s design with a commonly used term. The present title is suitable for a longitudinal study.
Introduction needs explanations for the necessity of the work. The rates of breastfeeding initiation and continuation in the country should be included in the introduction section.
PLS present key elements of study design in the methods section
PLS describe the setting and location including the number of deliveries and the type of hospital in which the data collected.
PLS describe any efforts to address potential sources of bias
PLS describe limitations of the study both in the abstract and in discussion.


[Note: HTML markup is below. Please do not edit.]

Reviewers' comments:

Reviewer's Responses to Questions

**Comments to the Author**

1. Is the manuscript technically sound, and do the data support the conclusions?

The manuscript must describe a technically sound piece of scientific research with data that supports the conclusions. Experiments must have been conducted rigorously, with appropriate controls, replication, and sample sizes. The conclusions must be drawn appropriately based on the data presented.

Reviewer #1: No

Reviewer #2: Yes

2. Has the statistical analysis been performed appropriately and rigorously?

Reviewer #1: Yes

Reviewer #2: Yes

3. Have the authors made all data underlying the findings in their manuscript fully available?

The [PLOS Data policy](http://www.plosone.org/static/policies.action#sharing) requires authors to make all data underlying the findings described in their manuscript fully available without restriction, with rare exception (please refer to the Data Availability Statement in the manuscript PDF file). The data should be provided as part of the manuscript or its supporting information, or deposited to a public repository. For example, in addition to summary statistics, the data points behind means, medians and variance measures should be available. If there are restrictions on publicly sharing data—e.g. participant privacy or use of data from a third party—those must be specified.

Reviewer #1: No

Reviewer #2: Yes

4. Is the manuscript presented in an intelligible fashion and written in standard English?

PLOS ONE does not copyedit accepted manuscripts, so the language in submitted articles must be clear, correct, and unambiguous. Any typographical or grammatical errors should be corrected at revision, so please note any specific errors here.

Reviewer #1: No

Reviewer #2: Yes

5. Review Comments to the Author

Please use the space provided to explain your answers to the questions above. You may also include additional comments for the author, including concerns about dual publication, research ethics, or publication ethics. (Please upload your review as an attachment if it exceeds 20,000 characters)

Reviewer #1: Thank you for your manuscript describing risk factors for mastitis to become a breast abscess. I have a few comments and queries:
1. Abstract- I don't think that your results as described lead to the conclusion that USS guided biopsy is essential - why not milk culture which is much less invasive?

Yes, editor, thank you for reminding me. This article was mainly a scientific analysis of mastitis progression to breast abscess risk factors. Ultrasound-guided puncture was used for the treatment of breast abscess, which had good results but should not be included in the conclusion of the abstract. I have deleted the relevant inappropriate conclusions.
2. Background - your background is important, I would emphasise the % of women who give up breastfeeding because of mastitis

In the background section, I added the importance of breastfeeding and the current situation of breastfeeding in China. I cited relevant literature to highlight mastitis and breast abscess as one of the important factors leading to the interruption of breastfeeding. It has been reported that about 10% of patients with mastitis or breast abscess will result in the interruption of breastfeeding.

3. Methods - How was the data collected? From hospital information systems or via hospital IDs? If so, the data was not anonymised directly. What was your denominator of deliveries, what type of hospital is the data collected from (high risk, normal etc.)

The retrospective longitudinal study was performed at Women and Children’s Hospital of Chongqing Medical University. It is a specialist general hospital for the treatment of women's and children's diseases. About 17,000 women give birth in our hospital every year. We collected data on all hospitalized patients diagnosed with mastitis or breast abscess between January 2019 and December 2020. The data were extracted from medical records and evaluated by two independent clinical physicians. The anonymity of medical data was strictly monitored by a doctor who had access to participants' information during and after data collection. To protect patient privacy, the data is anonymized, and the code does not include any information about the patient's identity.
4. Results - your data summary of risk factors with % would be better presented in a table. Also, you do not mention in your abstract that the majority of women are primiparous - which could mean that better breastfeeding advice is needed to avoid breast abscess. Similarly bacterial cultures would be better in a table.Did you test whether MSSA or MRSA had higher risk of abscess formation? You dont mention in your abstract that the majority of women would have received inadequate treatment because of a high rate of penicillin resistance. Would this not mean that guidelines for therapy need to be changed?

I have changed the format of the table to highlight %. Yes, most of our patients are primiparas. 80.1% in mastitis group and 84.9% in breast abscess group were primiparas. There was no statistically significant risk factor for mastitis progression to breast abscess at primiparas. But these patients should receive breastfeeding guidance and advice early in the disease to avoid mastitis and breast abscesses. However, our study was retrospective, and our data did not include whether patients received better breast-feeding advice. Maybe this is a good idea for future research. We ran some more tests about whether MSSA or MRSA had higher risk of abscess formation as your advice. Surprisingly, both univariate and multivariate analyses yielded statistically significant results. As a result, we have updated our conclusions. In the discussion section, we mentioned that Staphylococcus aureus is a common cause of mastitis and breast abscesses. We also concluded that it had high resistance to penicillin. Because staphylococcus aureus which is usually produced by β-lactamase, several scholars and guides also recommend that we should used penicillinase-resistant antibiotics, such as flucloxacillin to treat with mastitis or breast abscess.
5. Discussion - your discussion doesn't bring out the limitations to your study (these are in your conclusions but do'nt account for some of the factors above).

We added the limitations of the study in the discussion section.

Reviewer #2: 1. This study explored the risk factors associated with the development of a breast abscess due to breast mastitis and clarified that the formation of breast abscesses is multifactorial. The data is very detailed and well represented.

Thank you for your recognition of our work .
2. But the author mentioned little about the innovation of this work, which makes the study more like a mere validation of previous ones. It may be helpful to rewrite the introduction and Discussion sections.

In the background section, I added the importance of breastfeeding and the current situation of breastfeeding in China. I cited relevant literature to highlight mastitis and breast abscess as one of the important factors leading to the interruption of breastfeeding. We analyzed the bacterial culture results of milk and pus, and the difference of bacterial resistance, providing good advice for the treatment of mastitis and breast abscess. The results of this study may provide evidence-based information for the risk factors of mammary abscess during lactation in China, and help provide appropriate management advice, scientific prevention and treatment strategies and effective individualized care for the multidisciplinary team or related personnel involved in maternal and infant feeding management. We included these statements in the "Introduction" and "discussion" sections to show the significance and innovation of our research.
3.The diagnostic criteria of breast abscess are not very clear. Should non-echo areas and low echo areas be present simultaneously?

Thank you for reminding us that our previous statement was not accurate enough. We have revised the definition of breast abscess to make it clearer. Non-echo areas and low echo areas may exist separately when diagnosing breast abscess.

6. PLOS authors have the option to publish the peer review history of their article ([what does this mean?](https://journals.plos.org/plosone/s/editorial-and-peer-review-process#loc-peer-review-history)). If published, this will include your full peer review and any attached files.


If you choose “no”, your identity will remain anonymous but your review may still be made public.


**Do you want your identity to be public for this peer review?** For information about this choice, including consent withdrawal, please see our [Privacy Policy](https://www.plos.org/privacy-policy).

Reviewer #1: No

Reviewer #2: No

[NOTE: If reviewer comments were submitted as an attachment file, they will be attached to this email and accessible via the submission site. Please log into your account, locate the manuscript record, and check for the action link "View Attachments". If this link does not appear, there are no attachment files.]

While revising your submission, please upload your figure files to the Preflight Analysis and Conversion Engine (PACE) digital diagnostic tool, <https://pacev2.apexcovantage.com/>. PACE helps ensure that figures meet PLOS requirements. To use PACE, you must first register as a user. Registration is free. Then, login and navigate to the UPLOAD tab, where you will find detailed instructions on how to use the tool. If you encounter any issues or have any questions when using PACE, please email PLOS at [figures@plos.org](mailto:figures@plos.org). Please note that Supporting Information files do not need this step.
