# Supplementary material for: Risk factors and prognosis of acute lactation mastitis developing into a breast abscess: A retrospective longitudinal study in China
Source: PLoS One. 2022 Sep 1;17(9):e0273967. doi: 10.1371/journal.pone.0273967 (PMC9436116; doi:10.1371/journal.pone.0273967)
Supplement: Attachment — Submitted filename: Response to Reviewers.docx [file pone.0273967.s006.docx]

Response to Reviewers

PONE-D-21-29131R2

Risk factors and prognosis of acute lactation mastitis developing into a breast abscess：a retrospective longitudinal study in China.

PLOS ONE

Dear Dr. Gao,

Thank you for submitting your manuscript to PLOS ONE. After careful consideration, we feel that it has merit but does not fully meet PLOS ONE’s publication criteria as it currently stands. Therefore, we invite you to submit a revised version of the manuscript that addresses the points raised during the review process.
 
Please submit your revised manuscript by Sep 15 2022 11:59PM. If you will need more time than this to complete your revisions, please reply to this message or contact the journal office at [plosone@plos.org](mailto:plosone@plos.org). When you're ready to submit your revision, log on to https://www.editorialmanager.com/pone/ and select the 'Submissions Needing Revision' folder to locate your manuscript file.

Please include the following items when submitting your revised manuscript:

- A rebuttal letter that responds to each point raised by the academic editor and reviewer(s). You should upload this letter as a separate file labeled 'Response to Reviewers'.
- A marked-up copy of your manuscript that highlights changes made to the original version. You should upload this as a separate file labeled 'Revised Manuscript with Track Changes'.
- An unmarked version of your revised paper without tracked changes. You should upload this as a separate file labeled 'Manuscript'.

If you would like to make changes to your financial disclosure, please include your updated statement in your cover letter. Guidelines for resubmitting your figure files are available below the reviewer comments at the end of this letter.

If applicable, we recommend that you deposit your laboratory protocols in protocols.io to enhance the reproducibility of your results. Protocols.io assigns your protocol its own identifier (DOI) so that it can be cited independently in the future. For instructions see: <https://journals.plos.org/plosone/s/submission-guidelines#loc-laboratory-protocols>. Additionally, PLOS ONE offers an option for publishing peer-reviewed Lab Protocol articles, which describe protocols hosted on protocols.io. Read more information on sharing protocols at <https://plos.org/protocols?utm_medium=editorial-email&utm_source=authorletters&utm_campaign=protocols>.

We look forward to receiving your revised manuscript.

Kind regards,

Forough Mortazavi
Academic Editor
PLOS ONE

Journal Requirements:

Please review your reference list to ensure that it is complete and correct. If you have cited papers that have been retracted, please include the rationale for doing so in the manuscript text, or remove these references and replace them with relevant current references. Any changes to the reference list should be mentioned in the rebuttal letter that accompanies your revised manuscript. If you need to cite a retracted article, indicate the article’s retracted status in the References list and also include a citation and full reference for the retraction notice.

Additional Editor Comments (if provided):

Dear authors,
Thank you for submitting the revised manuscript. In order to move forward with this, however, we are requesting further revisions including:
1. In table 4, the reference group for the ‘white blood cell count’ variable should be identified.

Reference group for variable is the group of white blood cell count＜9.5×109/L. In Table4, we added annotations and identified reference groups. In the discussion section, we added explanations of statistical differences between univariate and multivariate analyses for routine blood counts.
2. Lines 83-85 should be revised.: [(3) a body temperature > 37.3℃; or (4) routine blood test results that showed increased white blood cells (WBCs) or neutrophils or increased C reactive protein levels. (4) Patients with positive milk culture.] PLS check the text again.

We checked the content and made corrections.
3. I noticed that in your responses to the reviewers’ comments you state, “It is a specialist general hospital for the treatment of women's and children's diseases. About 17,000 women give birth in our hospital every year.” This information should be stated in the methods section of the manuscript, too.
In the methods section, we have made changes as requested by the editor.

[Note: HTML markup is below. Please do not edit.]


[NOTE: If reviewer comments were submitted as an attachment file, they will be attached to this email and accessible via the submission site. Please log into your account, locate the manuscript record, and check for the action link "View Attachments". If this link does not appear, there are no attachment files.]

While revising your submission, please upload your figure files to the Preflight Analysis and Conversion Engine (PACE) digital diagnostic tool, <https://pacev2.apexcovantage.com/>. PACE helps ensure that figures meet PLOS requirements. To use PACE, you must first register as a user. Registration is free. Then, login and navigate to the UPLOAD tab, where you will find detailed instructions on how to use the tool. If you encounter any issues or have any questions when using PACE, please email PLOS at [figures@plos.org](mailto:figures@plos.org). Please note that Supporting Information files do not need this step.

*In compliance with data protection regulations, you may request that we remove your personal registration details at any time.* [*(Remove my information/details)*](https://www.editorialmanager.com/pone/login.asp?a=r)*. Please contact the publication office if you have any questions.*
